# Usefulness of digital infrared thermography video using FLIR T560 in detecting hypothermia associated with complex regional pain syndrome type I: A CARE-compliant case report

**DOI:** 10.1097/MD.0000000000041876

**Published:** 2025-06-20

**Authors:** Yewon Jang, Sungho Kim, Min Cheol Chang

**Affiliations:** a Department of Electronic Engineering, Yeungnam University, Gyeongsan-si, Republic of Korea; b Department of Physical Medicine and Rehabilitation, College of Medicine, Yeungnam University, Daegu, Republic of Korea.

**Keywords:** complex regional pain syndrome, hypothermia, pain, sensor, video

## Abstract

**Rationale:**

Because the diagnosis of complex regional pain syndrome (CRPS) majorly relies on patients’ subjective clinical complaints, CRPS can be overdiagnosed, underdiagnosed, or missed entirely in clinical practice. CRPS is related to dysfunction of the autonomic nervous system, which causes temperature changes in patients’ skin. This case report evaluates the effectiveness of the FLIR T560 portable thermal imaging camera in detecting hypothermia associated with CRPS type I, potentially enhancing diagnostic accuracy.

**Patient concerns:**

We present a 25-year-old female with pain and limited passive range of motion in all parts of her hands bilaterally for 7 months. The bilateral hand pain was aggravated by active and passive range of motion. Also, the patient had bilateral hyperesthesia and allodynia of her entire hands, and skin atrophy and hypohidrosis were observed on both hands. The skin color of both hands was relatively cyanotic. Stiffness was checked during the passive range of motion of her bilateral fingers. Additionally, mild motor weakness was checked on the finger flexor and extensor bilaterally.

**Diagnoses:**

The patient was diagnosed with CRPS type I. The FLIR T560 was used to record thermal images in an insulated room, revealing a lower surface temperature (approximately 1°C–2°C difference), shown as less bright, on her bilateral hands and forearms compared with a normal subject.

**Interventions:**

We administered Pregabalin 50 mg oral medication twice daily and the contrast bath.

**Outcomes:**

At 1-month-follow up, about 50% of her pain was reduced.

**Lessons:**

We demonstrate that the FLIR T560 thermal imaging camera can show hypothermia on the hands bilaterally in CRPS type I, which can be helpful for the accurate diagnosis of CRPS. The FLIR T560 camera is a portable and convenient tool that may support the diagnosis of CRPS.

## 1. Introduction

Complex regional pain syndrome (CRPS) is a clinical condition characterized by symptoms such as neuropathic pain, hypersensitivity, swelling, and disturbed autonomic and motor functions in the affected.^[1]^ CRPS can result from various causes including trauma, fractures, nerve injuries in the limbs, brain injuries, spinal cord injuries, heart attacks, or unknown factors.^[1]^ CRPS is frequently misunderstood and can be overdiagnosed, underdiagnosed, or missed entirely in clinical practice. Diagnosis relies on clinical symptoms, as the underlying pathophysiology is complex and not yet fully understood.

To diagnose CRPS based on objective findings rather than relying on subjective clinical findings, some diagnostic imaging tools have been developed.^[2]^ In clinical practice, 3-phase bone scintigraphy and digital infrared thermography imaging (DITI) are being used.^[3–6]^ The 3-phase bone scintigraphy test requires intravenous administration of a radiopharmaceutical and has the disadvantage of requiring a 3- to 4-hour waiting period after administration before imaging can be performed.^[7]^ Also, the DITI equipment has some practical drawbacks, particularly in terms of mobility. Most DITI setups rely on an external power source, and the whole system—including the camera, motorized stand, and console unit—is bulky and heavy. Consequently, when capturing temperature distribution data using DITI, patients must be transported to the imaging room, and a specific position and pose are required.

On the other hand, the FLIR T560 (FLIR, Seoul, Republic of Korea) thermal imaging camera used in our experiments is compact, light, and battery-powered, allowing us to record body temperature images conveniently in the examination room and easily adjust the operator based on the patient’s posture.^[8]^ The operation of this advanced thermal imaging camera is similar to that of DITI. Unlike DITI, which requires a computer for display, the FLIR T560 has a built-in display that allows for direct video playback on the device. This makes the FLIR T560 a suitable alternative to overcome the limitations of DITI. Table 1 compares the FLIR T560 thermal infrared camera to the IRIS-XP (Medicore, Hanam-si, Republic of Korea), a standard DITI instrument used in clinical practice.^[9,10]^

**Table 1:** Specifications of the IRIS-XP (digital infrared thermographic imaging) and FLIR T560.

	IRIS-XP	FLIR T560
IR resolution (pixels)	384 × 288 (640 × 480 is optional)	640 × 480
Frame rate (Hz)	9 or 30	30
Spectral range (μm)	8–14	7.5–14
Temperature measurement range (°C)	14.5~40	−20~120
Temperature sensitivity (°C)	0.05	0.04
Size (mm)	Camera + motorized stand: 460 × 550 × 155~1420, Console unit: 600 × 800 × 1330	Camera only: 140 × 201 × 167
Power supply method	Connect to a power outlet	Use battery
Compensation function of room temperature	O	O
Analytics software provided	O	O
PC system included	O	X
Portability	X	O

In this study, we report a case in which hypothermia associated with CRPS type 1 on the bilateral hands was detected using FLIR T560.

## 2. Case report

A 25-year-old woman visited the pain clinic of a university hospital due to pain and limited bilateral passive range of motion (ROM) over her entire hands bilaterally. Her symptoms began 7 months ago following a high-velocity thrust manipulation in a local pain clinic (numeric rating scale: 3; range: 0–10; 0: no pain, 10: worst pain imaginable), which was aggravated by active and passive ROM. The patient and her mother provided informed consent for participation in the study. The study was approved by the local Institutional Review Board of Yeungnam University Hospital.

Upon physical examination, the patient was found to have hyperesthesia and allodynia of her entire bilateral hands. Skin atrophy and hypohidrosis were observed on both hands. The skin color of her hands was relatively cyanotic bilaterally. Bilateral stiffness was checked during passive ROM of her fingers. Additionally, mild motor weakness was observed in her finger flexor and finger extensor (manual muscle test grade: 4). No abnormal findings were noted in a nerve conduction study and cervical spine magnetic resonance imaging. Based on the CRPS criteria proposed by the Budapest consensus group, we diagnosed the patient with CRPS type I.^[11]^

The FLIR T560, an infrared camera that functions within the long-wave infrared spectrum (7.5–14 μm), was employed to record videos displaying the distribution of the patient’s body temperature. The camera is capable of detecting temperature differences as slight as 0.04°C, with a detection range spanning from −20°C to 120°C and an accuracy margin of ±2°C.^[9]^ To maintain stable environmental conditions, the experiment was performed in a temperature-controlled room.^[12]^

Throughout the recording, the patient stood roughly 3 m away from the camera, rotating their body to provide clear views of the areas suspected of temperature anomalies. The patient rotated in 8 directions, pausing in each direction.

**Figure 1.**
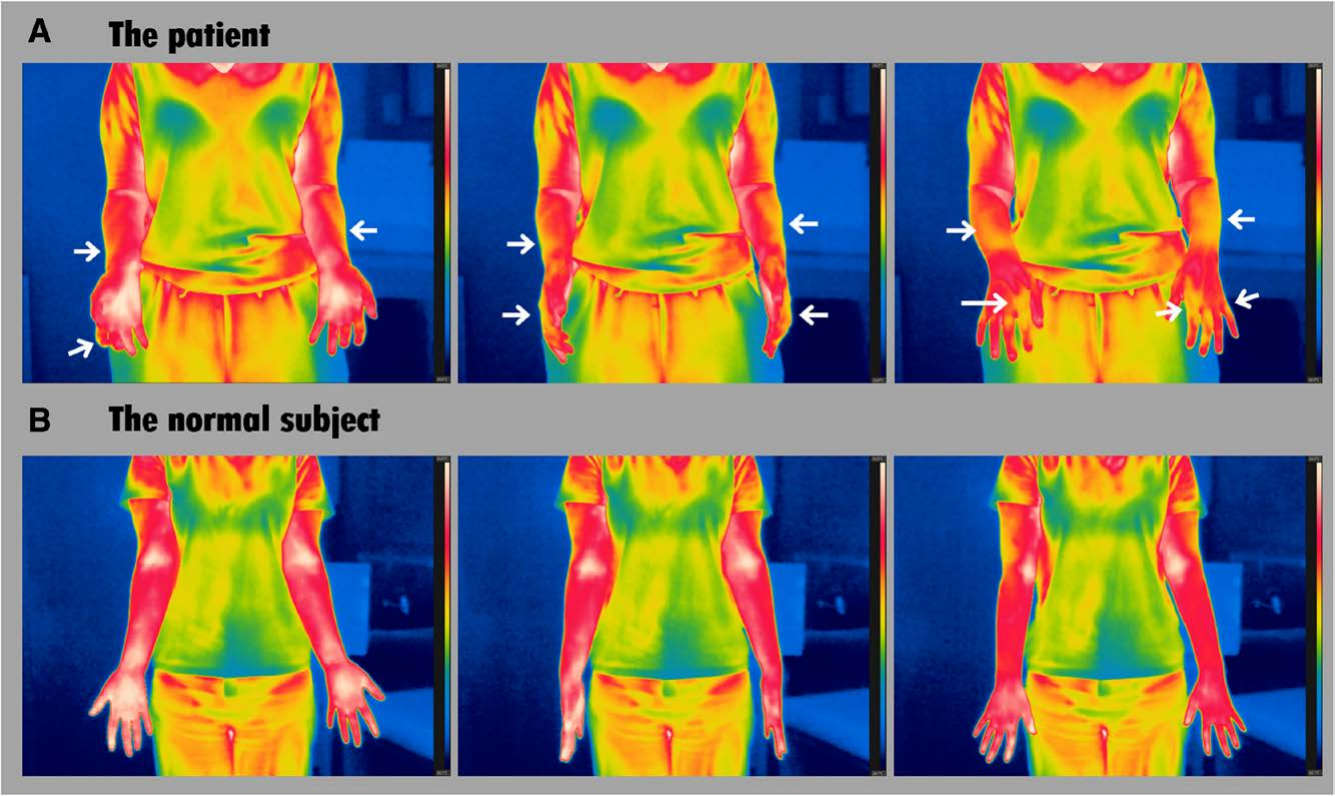
A 25-year-old woman (A) experiencing complex regional pain syndrome type I had a lower arm temperature (arrows) than a 24-year-old woman (B) who was asymptomatic.

To ensure precise infrared measurements, the camera’s calibration settings were adjusted as follows: ambient temperature at 25°C, humidity at 50%, distance set to 3 m, skin emissivity at 0.98, and surface reflection temperature at 25°C.^[8,12,13]^ The thermal video was recorded with the color mapping scale set between 24°C and 34°C, allowing for uniform temperature representation across all video frames. As a comparison for analyzing the patient’s temperature distribution video, a healthy individual (a 24-year-old woman) who has never experienced CRPS type I was recorded under the same environmental conditions. Sample frames extracted from the temperature distribution videos of the patient and the normal subject are shown in Figure 1.

According to the color bar displayed on the right side of each image, white, red, and brighter colors indicate higher surface temperatures, while blue and darker colors indicate lower temperatures (white color indicates the highest surface temperature). When comparing the body temperature color maps between the asymptomatic individual and the patient, in the normal subject, the color of the hands and forearms was predominantly white and red, but in the patient, there were relatively fewer white-colored areas on the hands and forearms, with yellow tones visible on the fingers and dorsum of the hands (Fig. 1) (see Supplemental Video, Supplemental Digital Content, https://links.lww.com/MD/P260, which demonstrates the video captured using the FLIR T560). These findings indicated that the patient’s hands and forearms were generally cooler than those of the normal subject. Additionally, while the forearm of the normal subject was uniformly red, the patient exhibited a considerable distribution of yellow on the dorsum and lateral sides of the forearm. The yellow regions indicated significantly lower temperatures compared with the white or red regions. These findings suggest that the patient’s forearm and hand were generally cooler than those of the normal subject, with an estimated temperature difference of approximately 1°C to 2°C.

We administered Pregabalin 50 mg oral medication twice daily and the contrast bath. At 1-month-follow up, about 50% of her pain was reduced, and this reduction was maintained even after 3 months.

## 3. Discussion

In this report, our patient with pain, limited passive ROM, and mild weakness in all parts of her hands bilaterally due to CRPS type I showed relatively fewer regions of white and red color on their bilateral hands and forearms on FLIR T560 compared with the normal subject. This finding indicates the patient’s hands and forearms were cooler than those of the normal subject. The lower temperature in the patient seems to be attributed to vasoconstriction or reduced sweat secretion following autonomic dysfunction induced by CRPS type I. CRPS is diagnosed based on clinical findings and patients’ symptoms, which is relatively subjective. The detection of body temperature changes using FLIR T560 can provide objectivity in diagnosing CRPS.

Most thermal infrared cameras operate by detecting and visualizing electromagnetic radiation emitted from objects with surface temperatures above 0 K (absolute temperature), following Planck law.^[8,14,15]^ Because the intensity of this radiation is proportional to temperature, these cameras can be used to observe temperature distributions and variations.^[8,14,15]^ Users can easily analyze temperature patterns by setting the type of color map, temperature range of color mapping, and environmental parameters. These systems are the same for the DITI and FLIR T560. However, the FLIR T560 is much more portable than the DITI. Its built-in display eliminates the need for an external personal computer or monitor, and its battery operation allows for the swift capture of temperature distribution images in clinical settings. This supports rapid image analysis and timely diagnosis.

Pain physicians can place the FLIR T560 device next to them while examining a patient and easily measure the patient’s skin temperature in real time. This provides the advantage of approximately identifying temperature changes by nerve lesions involving sympathetic dysfunction, such as CRPS. Also, since it captures video rather than just images, it offers the advantage of measuring the temperature of all areas of the body in real time, not just specific parts.

In conclusion, we report that the FLIR T560 thermal imaging camera can show skin temperature changes, which can be helpful for the accurate diagnosis of CRPS. In our opinion, the FLIR T560 thermal imaging camera can be a valuable tool for pain physicians, offering greater portability and convenience compared with traditional DITI systems. Additionally, it enables real-time measurement of skin temperature directly in the clinic, allowing rapid identification of whether a patient’s pain is associated with CRPS or not. The FLIR T560’s ability to display images on the device itself simplifies the diagnostic process by eliminating the need for additional equipment such as personal computers or monitors. Our study has some limitations. First, this is a single case study, and therefore, the clinical usefulness of the FLIR T560 for diagnosing CRPS cannot be definitely confirmed. Second, after treatment, the changes in thermal distribution on both forearms and hands were not evaluated using the FLIR T560. Third, the temperature was not measured quantitatively. Therefore, further studies to compensate for these limitations are required in the future.

## Author contributions

**Conceptualization:** Yewon Jang, Sungho Kim, Min Cheol Chang.

**Data curation:** Yewon Jang, Sungho Kim, Min Cheol Chang.

**Formal analysis:** Yewon Jang, Sungho Kim, Min Cheol Chang.

**Investigation:** Yewon Jang, Sungho Kim, Min Cheol Chang.

**Methodology:** Yewon Jang, Sungho Kim, Min Cheol Chang.

**Project administration:** Yewon Jang, Sungho Kim, Min Cheol Chang.

**Software:** Yewon Jang, Sungho Kim, Min Cheol Chang.

**Validation:** Yewon Jang, Sungho Kim, Min Cheol Chang.

**Visualization:** Yewon Jang, Sungho Kim, Min Cheol Chang.

**Writing – original draft:** Yewon Jang, Sungho Kim, Min Cheol Chang.

**Writing – review & editing:** Yewon Jang, Sungho Kim, Min Cheol Chang.

**Funding acquisition:** Sungho Kim, Min Cheol Chang.

**Resources:** Min Cheol Chang.

**Supervision:** Min Cheol Chang.

## Supplementary Material


